# Homoarginine and Progression of Chronic Kidney Disease: Results from the Mild to Moderate Kidney Disease Study

**DOI:** 10.1371/journal.pone.0063560

**Published:** 2013-05-15

**Authors:** Christiane Drechsler, Barbara Kollerits, Andreas Meinitzer, Winfried März, Eberhard Ritz, Paul König, Ulrich Neyer, Stefan Pilz, Christoph Wanner, Florian Kronenberg, for the MMKD Study Group

**Affiliations:** 1 Division of Nephrology, Department of Medicine, University of Würzburg, Würzburg, Germany; 2 Division of Genetic Epidemiology, Department of Medical Genetics, Molecular and Clinical Pharmacology, Innsbruck Medical University, Innsbruck, Austria; 3 Clinical Institute of Medical and Chemical Laboratory Diagnostics, Medical University of Graz, Graz, Austria; 4 Department of Public Health, Social and Preventive Medicine, Mannheim Medical Faculty, University of Heidelberg, Mannheim, Germany; 5 Department of Internal Medicine, Division of Nephrology, Ruprecht-Karls-University, Heidelberg, Germany; 6 Innsbruck University Hospital, Department of Clinical Nephrology, Innsbruck, Austria; 7 Department of Nephrology and Dialysis, Academic Teaching Hospital, Feldkirch, Austria; 8 Department of Epidemiology and Biostatistics, EMGO Institute for Health and Care Research, VU University Medical Centre, Amsterdam, The Netherlands; 9 Department of Internal Medicine, Division of Endocrinology and Metabolism, Medical University of Graz, Graz, Austria; German Diabetes Center, Leibniz Center for Diabetes Research at Heinrich Heine University Duesseldorf, Germany

## Abstract

**Background:**

Homoarginine is an amino acid derivative mainly synthesized in the kidney. It is suggested to increase nitric oxide availability, enhance endothelial function and to protect against cardiovascular diseases. We aimed to investigate the relation between homoarginine, kidney function and progression of chronic kidney disease (CKD).

**Methods:**

We measured plasma homoarginine concentrations in baseline samples of the Mild to Moderate Kidney Disease (MMKD) Study, a prospective cohort study of 227 patients with CKD in Europe. Homoarginine concentrations were available in 182 of the baseline samples and in 139 of the prospectively-followed patients. We correlated homoarginine concentrations to parameters of kidney function. The association between homoarginine and progression of CKD was assessed during a follow-up of up to seven years (median 4.45 years, interquartile range 2.54–5.19) using Cox regression analysis. Progression of CKD was defined as doubling of baseline serum creatinine and/or end-stage renal disease.

**Results:**

Study participants were at baseline on average 47±13 years old and 65% were male. Mean±standard deviation of homoarginine concentrations were 2.5±1.1 µmol/L and concentrations were incrementally lower at lower levels of GFR with mean concentrations of 2.90±1.02 µmol/L (GFR>90 ml/min), 2.64±1.06 µmol/L (GFR 60–90 ml/min), 2.52±1.24 µmol/L (GFR 30–60 ml/min) and 2.05±0.78 µmol/L (GFR<30 ml/min), respectively (p = 0.002). The age- and sex-adjusted risk to reach the renal endpoint was significantly higher by 62% with each decrease by one standard deviation (1.1 µmol/L) of homoarginine (HR 1.62, 95% CI 1.16–2.27, p = 0.005). This association was independent of proteinuria (HR 1.56, 95% CI 1.11–2.20, p = 0.01), and was slightly attenuated when adjusting for GFR (HR 1.40 (95% CI 0.98–1.98, p = 0.06).

**Conclusions:**

Homoarginine concentrations are directly correlated with kidney function and are significantly associated with the progression of CKD. Low homoarginine concentrations might be an early indicator of kidney failure and a potential target for the prevention of disease progression which needs further investigations.

## Introduction

Chronic kidney disease (CKD) represents a major public health problem with an increasing prevalence as well as an increase in the incidence rate of end-stage renal disease [1], [2]. The costs of treatment put an enormous burden on health care resources since renal replacement therapy represents one of the most expensive chronic therapies. Importantly, CKD per se has been shown to be a strong risk factor for cardiovascular morbidity and mortality [3]. Patients with a moderately impaired kidney function already have a high risk to develop cardiovascular complications [4]. Cardiovascular risk further increases with the decline in kidney function, and the majority of CKD patients die from cardiac and vascular events before reaching end-stage renal disease. Prevention of disease progression and associated complications therefore is highly important including the treatment of renal risk factors.

Homoarginine is a cationic amino acid, which is derived from lysine and mainly synthesized in the kidney by transaminidation of its precursor [5], [6]. Genetic studies identified polymorphisms of the L-arginine:glycine amidinotransferase (AGAT), which is the main enzyme responsible for homoarginine formation, to be significantly associated with kidney function in humans [7], [8]. Studies have shown that homoarginine may have beneficial effects on renal risk factors such as hypertension and glycemia. In an animal study, the administration of L-homoarginine increased urinary excretion of nitrate, the degradation product of NO, and reduced blood pressure in salt-sensitive hypertensive rats [9]. Homoarginine was found to stimulate insulin secretion [10], [11] which is relevant for the glycemic control to preserve kidney function [12]. Interestingly, disturbances in calcium-phosphate metabolism, which are known to contribute to the progression of CKD [13], are positively affected by homoarginine. Homoarginine was suggested to inhibit bone alkaline phosphatase [14], [15], negatively correlate to beta-crosslaps and osteocalcin and decrease the risk of fractures [16], [17]. Importantly, homoarginine serves as a precursor of NO [18]–[21] and inhibits arginase. Thus, homoarginine may increase the availability of NO and impede or ameliorate endothelial dysfunction [9], [18]–[22], which is crucial to prevent progression of CKD.

Therefore, we reasoned that a lack of homoarginine contributes to CKD progression: we hypothesized that a low blood concentration of homoarginine associates with a decline in kidney function and shorter time to end-stage renal disease. We therefore analyzed data of the Mild to Moderate Kidney Disease Study (MMKD Study), which is a prospective cohort study of 227 patients with primary nondiabetic CKD in Europe [23], [24].

## Methods

### Baseline Investigation

The methodology of the MMKD Study has previously been reported in detail [23], [24]. Briefly, the MMKD Study is a prospective cohort study including 227 white patients aged between 18 and 65 years with nondiabetic CKD and various degrees of kidney impairment. The patients were recruited from 8 nephrology departments in Germany, Austria, and South Tyrol (Italy) as previously described [25]. The patients had stable kidney function for at least 3 months before inclusion into the study. Exclusion criteria were serum creatinine >6 mg/dL (531 µmol/L), diabetes mellitus of any type, malignancy, liver, thyroid, or infectious diseases, nephrotic syndrome (defined as daily proteinuria >3.5 g/1.73 m^2^), organ transplantation, immunosuppressive treatment, allergy to ionic contrast media, treatment with fish oil or erythropoietin and pregnancy.

To avoid interobserver differences, all patients were recruited by a single investigator who visited all participating centers. Information on age, gender, smoking habits, comorbidities and antihypertensive treatment at baseline was recorded by patient interview and confirmed by checking patient records. A clinical examination completed the procedure. Body mass index (BMI) was calculated as weight (kg) divided by height (m) squared. Blood pressure was measured in sitting position. Hypertension was defined as BP>140/90 mmHg and/or the use of antihypertensive medication.

### Ethics Statement

The study was approved by the Institutional Ethic Committee of the University of Innsbruck, and all participants gave their informed consent before inclusion in the study.

### Prospective Follow-Up and Outcome Assessment

Patients were followed prospectively until the primary study endpoint or the end of the follow-up period was reached. The primary endpoint was defined as doubling of baseline serum creatinine and/or terminal renal failure necessitating renal replacement therapy (dialysis therapy or kidney transplantation). A total of 177 (78%) patients from the baseline cohort of 227 patients could be followed prospectively over a period of up to 7 years. Patients received regular follow-up care in the outpatient ward and the endpoint was assessed by medical record abstraction. Patients who were lost to follow-up (n = 50) had at baseline a significantly better renal function than patients who were not lost to follow-up (i.e., a higher mean GFR [91±44 versus 64±39 ml/min per 1.73 m^2^; P<0.01]). However, both groups did not differ significantly with respect to age and gender. Patients who were lost to follow-up had moved away or were not referred by their physicians for follow-up visits in the renal units.

### Homoarginine and GFR Measurement

Homoarginine was measured in plasma samples taken at baseline and stored at −80°C, using a reverse-phase HPLC method [26], [27]. Within-day coefficients of variation (CV) were 4.7% (for a control sample with 1.21 µmol/L) and 2.2% (3.53 µmol/L), and between-day CV were 7.9% (1.25 µmol/L) and 6.8% (3.66 µmol/L), respectively. GFR was assessed in all patients using the iohexol clearance technique as described in detail elsewhere [28].

### Statistical Analysis

Continuous variables were compared between various groups using unpaired t tests or the nonparametric Wilcoxon rank sum test in case of non-normally distributed variables. Continuous variables across the stages of CKD were analyzed using one-way ANOVA or Kruskal-Wallis test where appropriate. Dichotomized variables were compared using Pearson χ^2^-test. We explored the correlation of homoarginine with other parameters using either Pearson or Spearman correlation analysis depending on the distribution of the analyzed variables. Cox regression analyses were applied to calculate hazard ratios (HRs) and corresponding 95% confidence intervals per one standard deviation change of homoarginine for the progression to renal endpoints adjusted for age, sex, and other risk predictors of disease progression. The calculated Cox regression models did not violate the proportional hazards assumption. Moreover, using non-linear splines, models did not depart from linearity. A two-sided p-value<0.05 was considered statistically significant. Analyses were performed using SPSS for Windows version 18.0 and R 2.14.2.

## Results

### Patient Characteristics

Of all 227 non-nephrotic patients included into the MMKD study, 182 patients with biological material available had a homoarginine measurement in the baseline samples. The mean homoarginine concentration was 2.57±1.09 µmol/L. Characteristics of patients with and without homoarginine values were not significantly different (Table S1). The baseline clinical characteristics and laboratory data of these patients are reported in Table 1. Overall, patients had a mean age of 46±13 years and 67% were male. Of the 182 patients, 59 patients had CKD stage 1 with a GFR ≥90 ml/min, 35 patients had stage 2 with a GFR ≥60–89 ml/min, 51 patients had stage 3 with a GFR ≥30–59 ml/min and 37 patients had CKD stage 4 or 5 with a GFR<30 ml/min. Homoarginine concentrations were incrementally lower at lower levels of GFR with mean concentrations of 2.90±1.02 µmol/L (stage 1), 2.64±1.06 µmol/L (stage 2), 2.52±1.24 µmol/L (in stage 3) and 2.05±0.78 (stage 4–5), respectively. The differences in mean homoarginine concentrations across the stages of CKD were highly significant (p = 0.002). By correlation analyses, homoarginine concentrations were significantly related to GFR (r = 0.25, p = 0.001), proteinuria (r = −0.21, p = 0.005) and creatinine (r = −0.31, p<0.001). Homoarginine concentrations did not correlate with age (r = −0.06, p = 0.44), but slightly with BMI (r = 0.17, p = 0.02) and albumin (r = 0.15, p = 0.05), respectively. Patients with a lower GFR were older, had a higher BMI and lower serum albumin concentrations than those with a higher GFR.

**Table 1:** Baseline clinical and laboratory data of 182 patients with non-diabetic chronic kidney disease stratified by GFR stages according to K/DOQI guidelines.

		GFR (mL/min/1.73 m^2^)				
		≥90	60–89	30–59	<30	
Variable	all patients	(n = 59)	(n = 35)	(n = 51)	(n = 37)	p-value
Sex: males/females, n (%)	122/60	41/18	24/11	35/16	22/15	0.75
	(67.0/33.0)	(69.5/30.5)	(68.6/31.4)	(68.6/31.4)	(59.5/40.5)	
Age (years)	45.8±12.8	40.5±13.4	45.8±12.3	45.9±11.7	54.2±9.0	<0.001
BMI (kg/m^2^)	25.21±3.6	24.2±3.3	25.8±3.6	25.1±3.1	26.2±4.3	0.04
Current smokers, n (%)	36 (20)	15 (25)	8 (23)	6 (12)	7 (19)	0.73
Systolic blood pressure (mmHg)	137±21	135±22	138±25	138±18	138±19	0.82
Diastolic blood pressure (mmHg)	86±13	83±13	86±13	86±13	88±14	0.40
Serum albumin (g/dL)	4.6±0.4	4.7±0.4	4.4±0.6	4.6±0.4	4.5±0.4	0.005
Proteinuria (g/24 h/1.73 m^2^)	0.90±0.90	0.56±0.65	1.10±1.11	1.01±0.95	1.10±0.81	0.001
	(0.18;0.55;1.26)	(0.12;.0.35;0.73)	(0.17;0.60;1.80)	(0.22;0.55;1.78)	(0.54;0.95;1.52)	
GFR (mL/min/1.73 m^2^)	69±43	120±29	73±9	45±7	19±8	<0.001
	(38;63;96)	(96;111;134)	(65;70;81)	(40;44;50)	(12;18;27)	
Creatinine - standardized	179±113	89±21	136±49	202±72	334±115	<0.001
measurement (µmol/L)	(96;135;231)	(73;84;107)	(108;127;142)	(154;188;237)	253;319;422)	
Homoarginine (µM/L)	2.57±1.09	2.90±1.02	2.64±1.06	2.52±1.24	2.05±0.78	0.002

GFR denotes glomerular filtration rate measured by iohexol clearance, BMI; body-mass index.

Data are presented as mean ± SD and 25^th^, 50^th^ (median) and 75^th^ percentiles for skewed variables where appropriate.

P-values are for comparison across all four groups obtained from Kruskal-Wallis test, one-way ANOVA and χ^2^ test where appropriate.

### Homoarginine and Progression of CKD

Of the 177 patients in the MMKD study who could be followed prospectively until the end of the study or occurrence of the primary renal endpoint, a total of 139 patients had a homoarginine measurement available at baseline. Patients with and without homoarginine measurements and follow-up time were very similar in their clinical characteristics (Table S2). The median follow-up time was 4.45 years with an interquartile range of 2.54–5.19 years. Of those 139 patients, 56 patients (40.3%) experienced a renal disease progression (doubling of baseline serum creatinine and/or terminal renal failure necessitating renal replacement therapy). Homoarginine concentrations were significantly lower in these patients as compared to those without kidney disease progression (2.19±0.93 versus 2.71±1.13 µmol/L, respectively, p = 0.005). The further characteristics of the patients with and without kidney disease progression are presented in Table 2.

**Table 2:** Baseline clinical and laboratory data of the 139 patients who completed follow-up and stratified by patient groups with and without progression of chronic kidney disease.

	All patients	Non-progressors	Progressors	
Variable	(n = 139)	(n = 83)	(n = 56)	*P*-value^a^
Sex: males/females, n (%)	90/49	54/29	36/20	0.93
	(64.7/35.3)	(65.1/34.9)	(64.3/35.7)	
Age (years)	46.6±12.5	45.2±13.0	48.6±11.4	0.18
BMI (kg/m^2^)	25.3±3.6	25.0±3.5	25.7±3.8	0.22
Current smokers, n (%)	22 (15.8)	11 (13.3)	11 (19.6)	0.32
Systolic blood pressure (mmHg)	136±20	135±22	138±17	0.32
Diastolic blood pressure (mmHg)	85±12	84±13	88±12	0.09
Serum albumin (g/dL)	4.6±0.4	4.6±0.5	4.6±0.4	0.99
Proteinuria (g/24 h/1.73 m^2^)	1.00±0.92	0.80±0.93	1.30±0.84	<0.001
	(0.24;0.69;1.54)	(0.14;0.36;1.14)	(0.63;1.10;1.85)	
GFR (mL/min/1.73 m^2^)	62±41	79±41	37±24	<0.001
	(34;52;87)	(50;70;100)	(19;33;45)	
Creatinine (µmol/L)	195±118	131±63	289±119	<0.001
	(105;157;253)	(90;119;158)	(194;281;385)	
Homoarginine (µM/L)	2.50±1.08	2.71±1.13	2.19±0.93	0.005

GFR denotes glomerular filtration rate measured by iohexol clearance, BMI; body-mass index.

Data are presented as mean ± SD and 25^th^, 50^th^ (median) and 75^th^ percentiles for skewed variables where appropriate.

a P value for comparison between progressors and non-progressors.

We performed Cox regression analyses considering the time of reaching the kidney endpoint (Table 3). The age and sex-adjusted hazard ratio to suffer a kidney endpoint was significantly higher by 62% with each decrease of one standard deviation (1.1 µmol/L) of homoarginine (HR 1.62, 95% CI 1.16–2.27, p = 0.005). Further adjustment for proteinuria did not change the results (HR 1.56, 95% CI 1.11–2.20, p = 0.01). Additional adjustment for measured GFR slightly attenuated the association, resulting in a borderline significant hazard ratio of 1.40 (95% CI 0.98–1.98, p = 0.06).

**Table 3:** The association of homoarginine with progression of kidney disease during the observation period using multiple Cox proportional hazards regression models.

	Model 1^a^		Model 2^a^		Model 3^a^	
Variable (1 SD decrement)	HR (95% CI)	p	HR (95% CI)	p	HR (95% CI)	p
Homoarginine (−1.1 µM/L)	1.62 (1.16–2.27)	0.005	1.56 (1.11–2.20)	0.010	1.40 (0.98–1.98)	0.06
Proteinuria (0.92 g/24 h/1.73 m^2^)	–	–	1.32 (1.05–1.67)	0.019	1.36 (1.06–1.75)	0.016
GFR (−41 mL/min/1.73 m^2^)	–	–	–	–	4.98 (1.98–8.85)	<0.001

The hazard ratios (HR) and 95% confidence intervals (CI) were determined by univariate and multiple Cox proportional hazards regression analysis and are indicated for each decrement of 1 standard deviation (SD). For proteinuria, hazard ratios are indicated for each increment of 1 SD.

a The estimates in model 1 are adjusted for age and sex, those in model 2 are adjusted for age, sex and proteinuria and those in model 3 are adjusted for age, sex, proteinuria and GFR.

In additional analyses we investigated the role of nutritional status in the association of homoarginine with the progression of CKD. Neither BMI nor albumin was associated with the progression of kidney disease and the relationship of homoarginine with CKD progression was still significant after adjustments for albumin and BMI (HR 1.44, 95% CI 1.01–2.05, p = 0.045).

## Discussion

This is the first study that describes plasma homoarginine concentrations in patients with primary non-diabetic chronic kidney disease not requiring dialysis. The main findings of this study are that 1) circulating homoarginine concentrations in CKD patients are significantly lower with lower GFR; 2) homoarginine concentrations were substantially lower in patients with kidney disease progression as compared to those without progression; 3) circulating homoarginine concentrations are inversely associated with the risk to reach a kidney endpoint, independently of age, sex and proteinuria. This association was slightly attenuated after additional adjustment for GFR (p = 0.06).

Interestingly, up to now all parameters investigated in the MMKD Study showed a correlation of high concentrations with impaired kidney function as well as progression of CKD which points at a (direct or indirect) role of the kidney in their elimination [24], [25], [29]. Homoarginine is the first parameter in the MMKD Study, which showed not only lower concentrations at decreased kidney function, but also a higher probability of CKD progression at low concentrations. This is in line with the finding that homoarginine is mainly synthesized in the kidney by transaminidation of lysine [6]. The formation of homoarginine in the kidney is mediated by the l-arginine:glycine amidinotransferase (AGAT or GATM). It has been shown that there is an organ-specific pattern of AGAT expression with highest levels in renal tissue, which supports a crucial role of the kidney in homoarginine metabolism [30]. This enzyme also catalyzes an essential step of the creatine metabolism [31], namely the conversion of arginine and glycine into guanidinoacetate and ornithine. It is a reversible process which depends on the concentration of co-reactants. If lysine is sufficiently available, AGAT converts lysine together with guanidinoacetate to form homoarginine (and glycine) [5]. Since AGAT is able to catalyze a number of transamidination reactions, homoarginine can also be formed from other substrates, e.g. by the AGAT-mediated reaction of guanidinopropionic acid with ornithine. AGAT is mainly present in the kidney, but also in various other organs including the liver, the pancreas and the heart. The importance of the kidney for homoarginine synthesis is supported by our observation of a virtually linear association between homoarginine concentrations and GFR. In our cross-sectional analyses, we found homoarginine concentrations lower at lower levels of GFR. These findings are entirely consistent with genome-wide association studies (GWAS) showing that polymorphisms of AGAT are significantly associated with GFR [7], [8].

In line with this, homoarginine is believed to derive mainly from endogeneous synthesis rather than from nutrition: Although it cannot be fully excluded that uptake from the diet contributes to normal endogenous homoarginine levels [32], diet is unlikely to form a meaningful source of homoarginine status. Only small amounts of homoarginine are found in lentil and similar legumes. Noteworthy concentrations are exclusively present in two plant species of the grass pea family Lathyrus cicera [33] and Lathyrus sativus [34] which are not part of a usual Western diet. We are also aware that protein-energy wasting which frequently develops in patients with CKD might contribute to a decline in kidney function. The correlation of homoarginine with BMI and albumin as parameters of nutritional status, however, were only moderate. In our additional analyses, neither BMI nor albumin were associated with the progression of kidney disease and it is important to note that the relationship of homoarginine with progression was still significant after adjustments for albumin and BMI. Our results therefore support the notion that homoarginine concentration and its effect upon clinical outcome is not meaningfully affected by nutrition.

Our finding of an association of homoarginine with kidney function is furthermore in line with previous clinical studies. In the LURIC cohort comprising patients undergoing coronary angiography, homoarginine was significantly correlated to GFR with a correlation coefficient of 0.23 (p<0.001). The mean homoarginine concentration in this cohort was 2.6±1.1 µmol/L and patients had a mean GFR of 81±19 ml/min [35]. Of note, homoarginine concentrations were less than half as high in patients with end-stage renal disease requiring maintenance dialysis: patients in the 4D study had a mean homoarginine concentration of 1.2±0.5 µmol/l [35]. The concentrations that identified the respective interquartile ranges in the two cohorts were meaningfully lower in the 4D as compared to the LURIC study (0.87–1.4 µmol/L versus 1.85–3.1 µmol/L). The interquartile range for homoarginine in the MMKD Study was 1.81–3.13 µmol/L, which is close to that found in the LURIC study. Patients in the present study had mild to moderate kidney failure and a mean GFR of 69±43 ml/min. The main explanation for the decreased homoarginine concentrations in patients with advanced stages of kidney disease might be due to reduced activity of AGAT (see above). This hypothesis is supported by an experimental study by Tofuku et al. who found a decreased renal AGAT activity in rabbits with chronic kidney failure [36]. Similarly, plasma concentrations of homoarginine were significantly decreased in nephrectomized rats as compared to a sham-operated control group [37]. Taken together, low homoarginine concentration may therefore be an early indicator of kidney failure likely reflecting a decreasing synthesis capacity of the kidney [5].

In a second and independent synthesis route, homoarginine can be formed within an alternative urea cycle. Through this cycle, homoarginine serves as a precursor of NO by increasing the intracellular concentration of L-arginine, which is the main substrate for NO synthase [18]–[21]. Homoarginine is also an inhibitor of arginase, further increasing the bioavailability of arginine and enhancing nitric oxide formation. Thus, homoarginine may increase the availability of NO and impede or ameliorate endothelial dysfunction [9], [18]–[22], which is crucial to prevent progression of CKD. In previous studies, low homoarginine was correlated to markers of impaired endothelial function, i.e. ICAM-1 and VCAM-1 [35]. It may therefore be speculated whether lack of homoarginine is not only a risk marker, but potentially a risk factor in the progression of CKD. In our study, low homoarginine was significantly associated with disease progression, which was independent of age, sex and proteinuria and which was slightly attenuated when adjusted for baseline GFR. However, adjustment for GFR could be considered as an overadjustment since homoarginine is significantly associated with GFR and may even mediate the effect of lower GFR on progression of kidney disease leading hypothetically to a vicious cycle.

The previously shown experimental effects of homoarginine on blood pressure regulation and calcium-phosphate metabolism may represent further pathways by which homoarginine could potentially affect the course of kidney function. Previous studies found that administration of L-homoarginine increased urinary excretion of nitrate, the degradation product of NO, and reduced blood pressure in salt-sensitive hypertensive rats [9]. Homoarginine is known to be an inhibitor of bone alkaline phosphatase [14], [15] and was suggested to negatively correlate to beta-crosslaps, osteocalcin and decrease the risk of fractures. In addition, experimental studies showed that homoarginine increases insulin secretion and inhibits platelet aggregation [10], [11], [38]. In summary, these reports support the findings of our study and suggest mechanisms, by which homoarginine may preserve kidney function in the reduction of renal risk factors [10], [11], [14], [15], [18]–[21], [38].

### Strengths and Limitations of the Study

We consider it a strength of our study that GFR was not calculated by a formula but was measured by iohexol clearance which is considered an exact method to measure kidney function. In this context we refrained from investigating creatinine and cystatin C as surrogates of renal function, given that important novel findings pointed out the non-GFR related effects of these parameters [39].

Potential limitations of our study deserve also comments. Due to the observational design of our study, we cannot prove causality of the associations between homoarginine, kidney function and kidney disease progression. Furthermore, our study is limited by the sample size, and homoarginine measurements were not available in all patients of the MMKD Study. The lack of material in 38 patients out of 177 followed did not produce a particular selection bias: these 38 patients without homoarginine measurement were not different in major risk factors compared to those with measurements available (see Table S2). Finally, since we investigated only patients of Caucasian origin, it has to be shown whether our findings can be confirmed in different ethnic groups.

## Conclusion

We have found homoarginine concentrations directly correlated with kidney function in patients with CKD. Furthermore, low homoarginine concentrations were significantly associated with the progression of kidney disease. Low homoarginine concentrations may be an early indicator of kidney failure and potentially useful as a marker of disease progression. Whether homoarginine metabolism is causally relevant for kidney disease progression deserves further studies including randomized controlled trials with homoarginine supplementation.

## Supporting Information

Table S1 **Baseline clinical and laboratory data of 182 patients with homoarginine measurements available vs. 45 patients without measurements.**
(PDF)

Table S2 **Baseline clinical and laboratory data of the 177 patients who completed the follow-up.** The patient group is stratified to 139 patients with homoarginine measurements available vs. 38 patients without measurements.
(PDF)
