# Supplementary material for: Homoarginine and Progression of Chronic Kidney Disease: Results from the Mild to Moderate Kidney Disease Study
Source: PLoS One. 2013 May 15;8(5):e63560. doi: 10.1371/journal.pone.0063560 (PMC3655120; doi:10.1371/journal.pone.0063560)
Supplement: Table S1 — Baseline clinical and laboratory data of 182 patients with homoarginine measurements available vs. 45 patients without measurements. (PDF) [file pone.0063560.s001.pdf]

Table S1. Baseline clinical and laboratory data of 182 patients with homoarginine measurements available vs. 45 patients without measurements.

**Variable**	**With homoarginine available (n=182)**	**Without homoarginine (n=45)**	***P*-value**
Sex: males/females, n (%)	122/60 (67.0/33.0)	32/13 (71.1/28.9)	0.60
Age (years)	45.8±12.8	45.0±11.6	0.52
BMI (kg/m²)	25.1±3.6	25.2±4.5	
Current smokers, n (%)	36 (20)	13 (29)	0.36
Systolic blood pressure (mmHg)	137.0±20.7 (120;135;150)	138.5±21.2 (120;140;155)	0.67
Diastolic blood pressure (mmHg)	85.5±13.0	91.4±16.3	0.01
Serum albumin (g/dL)	4.6±0.4	4.6±0.4	0.73
Proteinuria (g/24h/1.73 m²)	0.90±0.90 (0.18;0.55;1.26)	1.01±0.91 (0.17;0.81;1.63)	0.43
GFR (mL/min/1.73m²)	69±43 (38;63;96)	71±37 (39;69;93)	0.66
Creatinine (μmol/L)	179±113 (96;135;231)	172±101 (105;129;193)	0.94
