# Supplementary material for: Homoarginine and Progression of Chronic Kidney Disease: Results from the Mild to Moderate Kidney Disease Study
Source: PLoS One. 2013 May 15;8(5):e63560. doi: 10.1371/journal.pone.0063560 (PMC3655120; doi:10.1371/journal.pone.0063560)
Supplement: Table S2 — Baseline clinical and laboratory data of the 177 patients who completed the follow-up. The patient group is stratified to 139 patients with homoarginine measurements available vs. 38 patients without measurements. (PDF) [file pone.0063560.s002.pdf]

Table S2. Baseline clinical and laboratory data of the 177 patients who completed the follow-up. The patient group is stratified to 139 patients with homoarginine measurements available vs. 38 patients without measurements.

**Variable**	**With homoarginine available (n=139)**	**Without homoarginine (n=38)**	***P*-value**
Sex: males/females, n (%)	90/49 (64.7/35.3)	28/10 (73.7/26.3)	0.30
Age (years)	46.6±12.5	45.6±11.3	0.46
BMI (kg/m²)	25.3±3.6	24.8±3.9	0.49
Current smokers, n (%)	22 (16)	12 (32)	0.08
Systolic blood pressure (mmHg)	136.2±20.2	138.5±19.9	0.53
Diastolic blood pressure (mmHg)	85.4±12.5	91.0±14.9	0.02
Serum albumin (g/dL)	4.6±0.4	4.6±0.3	0.82
Proteinuria (g/24h/1.73 m²)	1.00±0.92 (0.24;0.69;1.54)	1.04±0.92 (0.16;0.84;1.77)	0.87
GFR (mL/min/1.73m²)	62±41 (34;52;87)	69±34 (39;70;92)	0.15
Creatinine (μmol/L)	195±118 (105;157;253)	174±106 (105;132;189)	0.41
